# Supplementary material for: Direct Interaction of Avian Cryptochrome 4 with a Cone Specific G-Protein
Source: Cells. 2022 Jun 27;11(13):2043. doi: 10.3390/cells11132043 (PMC9265643; doi:10.3390/cells11132043)
Supplement: Supplementary file 1 [file cells-11-02043-s001.zip › cells-1774822-supplementary.pdf]

Supplementary information

Direct interaction of avian cryptochrome 4 with a cone specific G-protein

Katharina Görtemaker[1,*], Chad Yee[1,*], Rabea Bartölke[2,*], Heide Behrmann[3], Jan-Oliver Voß[1], Jessica Schmidt[2], Jingjing Xu[2], Vita Solovyeva[4], Bo Leberecht[2], Elmar Behrmann[3], Henrik Mouritsen[2,5] and Karl-Wilhelm Koch[1,5,#]

[1]Department of Neuroscience, Division of Biochemistry, University of Oldenburg, D-26111 Oldenburg, Germany;

[2]Department of Biology and Environmental Sciences, Neurosensorics/Animal Navigation, University of Oldenburg, D-26111 Oldenburg, Germany;

[3]Institute of Biochemistry, Faculty of Mathematics and Natural Sciences, University of Cologne, D-50674 Cologne, Germany

[4]Institute of Physics, University of Oldenburg, D-26111 Oldenburg, Germany

[5]Research Center for Neurosensory Sciences, University of Oldenburg, 26111 Oldenburg, Germany.

* These authors contributed equally to the work

# to whom correspondence should be addressed:

[1]Department of Neuroscience, Division of Biochemistry, University of Oldenburg, 26111 Oldenburg, Germany; Tel: +49 0441 798 3640; e-mail: karl.w.koch@uni-oldenburg.de

```
CLUSTAL O(1.2.4) multiple sequence alignment


ErGtα           ------------------------------------------------------------   0
BtGαi1          MLKRPGLVFFRFPTLAALPPAPQGAGPHTSLGVSKREEIITLSSRKPARRTSRERQPAFA   60


ErGtα           ---MGSGASAEDKEMAKRSKELEKKLQEDADKEAKTVKLLLLGAGESGKSTIVKQMKIIH   57
BtGαi1          PGTMGCTLSAEDKAAVERSKMIDRNLREDGEKAAREVKLLLLGAGESGKSTIVKQMKIIH   120
                   **.  *****  .:*** ::::*:**.:* *: ************************

ErGtα           QDGYTKEECMEFKSIIYGNILQSILAIIRAMSTLGIDYAESSCADEGRLLFNLADSIEEG   117
BtGαi1          EAGYSEEECKQYKAVVYSNTIQSIIAIIRAMGRLKIDFGDSARADDARQLFVLAGAAEEG   180
                : **::*** ::*:::*.* :***:******. * **:.:*: **:.* ** **.: ***

ErGtα           TMPPELVNCIKKLWKDGGVQACFDRAAEYQLNDSAAYYLNQLDRITAANYLPNEQDVLRS   177
BtGαi1          FMTAELAGVIKRLWKDSGVQACFNRSREYQLNDSAAYYLNDLDRIAQPNYIPTQQDVLRT   240
                 *  **.. **:****.******:*: *************:****:  **:*.:*****:

ErGtα           RVKTTGIIETKFSVKDLNFRMFDVGGQRSERKKWIHCFEGVTCIIFCGALSAYDMVLVED   237
BtGαi1          RVKTTGIVETHFTFKDLHFKMFDVGGQRSERKKWIHCFEGVTAIIFCVALSDYDLVLAED   300
                *******:**:*:.***:*:**********************.**** *** **:**.**

ErGtα           DEVNRMHESLHLFNSICNHKFFAATSIILFLNKKDLFEEKIKKVHLSICFPEYDGPNTFE   297
BtGαi1          EEMNRMHESMKLFDSICNNKWFTDTSIILFLNKKDLFEEKIKKSPLTICYPEYAGSNTYE   360
                :*:******::**:****:*:*: *******************  *:**:*** * **:*

ErGtα           DAGNYIKTQFLDLNMRKDVKEIYSHMTCATDTQNVKFVFDAVTDVIIKENLKDCGLF      354
BtGαi1          EAAAYIQCQFEDLNKRKDTKEIYTHFTCATDTKNVQFVFDAVTDVIIKNNLKDCGLF      417
                :*. **: ** *** ***.****:*:******:**:************:********
```

Figure S1. Sequence alignment. European robin gene *ErGNAT2* encoding ErGtα and the inhibitory G protein α-subunit type 1 from Bovine taurus (BtGαi1) are compared. In ErGtα amino acids 220 to 298 were replaced with the corresponding Bovine sequence Giα-1 (amino acids 283 to 361). The region is highlighted in yellow.

```
ATGGGGAGCGGGGCCAGTGCTGAGGACAAGGAGATGGCCAAGAGGTCCAAGGAGCTGGAGAAGAAGCTCCAG
GAAGATGCGGATAAGGAGGCCAAGACAGTCAAGTTGCTGCTGCTTGGTAAGGCTGGAG
                                              GG - - -GGCTGGAGAGTCAGGGAAGAGCA
CCATCGTGAAGCAGATGAAGATCATCCACCAGGACGGTTACACGAAGGAGGAGTGCATGGAGTTCAAGTCCATCA
TCTATGGCAACATCCTGCAGTCCATCCTGGCCATCATCCGCGCCATGTCCACGCTGGGCATCGACTACGCCGAGTC
CTCCTG-CGC-GG
  CCTGTCGCAGGATGAAGGCCGGCTGCTGTTCAACCTGGCTGACTCCATCGAGGAGGGCACCATGCCCCCCGA
GCTGGTGAACTGCATCAAGAAGCTGTGGAAGGATGGGGGGGTCCAGGCGTGCTTTGACCGCGCTGCCGAGTATC
AGCTCAACGACTCAGCTGCGTATTACCTGAACCAGCTGGACAGGATCACAGCTGCCAACTACCTCCCCAACGAGCA
GGACGTGCTGCGATCCCGAGTGAAGACCACAGGGATCATCGAGACCAAGTTCTCTGTCAAAGACCTGAATTT
CAGG -TGTGTG
CAGGATGTTTGACGTGGGAGGGCAGCGCTCAGAGAGGAAGAAGTGGATCCACTGCTTCGAGGGGGTGACCTGC
ATCATCTTCTGCGGGGCCCTGAGCGCCTACGACATGGTGCTGGTGGAGGATGATGAAGTG
                                                            GAACCGGATGCATGA
ATCCCTGCACCTATTCAACAGTATATGCAACCACAAGTTCTTTGCTGCCACCTCCATCATCCTCTTCCTCAACAAGAA
GGACCTTTTTGAGGAAAAGATCAAGAAAGTTCATCTCAGCATCTGCTTCCCAGAGTATGATGGT
                                                              GGTCCAAACACGTTT
GAGGACGCAGGGAATTACATCAAGACCCAGTTCCTGGACCTCAACATGAGGAAGGATGTGAAGGAGATCTACAG
CCACATGACCTGTGCCACAGACACGCAGAACGTCAAGTTCGTGTTCGACGCCGTCACAGACGTGATCATCAAAGA
GAACCTCAAGGACTGTGGCCTCTTCTGA
```

Figure S2. *Er*GNAT2 cDNA sequence (coding for cone specific Gα-subunit) reconstruction from *Erithacus rubecula* (blastn). Known GNAT2 sequences from different bird species [*Ficedula albicollis* (collared flycatcher), *Taeniopygia guttata* (zebra finch) and *Gallus gallus* (chicken)] were used to search the robin (*Erithacus rubecula)* genome from the Bird 10.000 genomes (B10K) Project (PRJNA545868) (S Feng S et al. Dense sampling of bird diversity increases power of comparative genomics. Nature 587, 252-257 (2020), ref. [49]. The cDNA was reconstructed from these exons as shown, with overlapping ends highlighted in yellow.

```
CLUSTAL O(1.2.4) multiple sequence alignment


genome ATGGGGAGCGGGGCCAGTGCTGAGGACAAGGAGATGGCCAAGAGGTCCAAGGAGCTGGAG     60
clone  ATGGGGAGCGGGGCCAGTGCTGAGGACAAGGAGATGGCCAAGAGGTCCAAGGAGCTGGAG     60
       ************************************************************

genome AAGAAGCTCCAGGAAGATGCGGATAAGGAGGCCAAGACAGTCAAGTTGCTGCTGCTTGGG     120
clone  AAGAAGCTCCAGGAAGATGCGGATAAGGAGGCCAAGACAGTCAAGTTGCTGCTGCTTGGG     120
       ************************************************************

genome GCTGGAGAGTCAGGGAAGAGCACCATCGTGAAGCAGATGAAGATCATCCACCAGGACGGT     180
clone  GCTGGAGAGTCAGGGAAGAGCACCATCGTGAAGCAGATGAAGATCATCCACCAGGACGGT     180
       ************************************************************

genome TACACGAAGGAGGAGTGCATGGAGTTCAAGTCCATCATCTATGGCAACATCCTGCAGTCC     240
clone  TACACGAAGGAGGAGTGCATGGAGTTCAAGTCCATCATCTATGGCAACATCCTGCAGTCC     240
       ************************************************************

genome ATCCTGGCCATCATCCGCGCCATGTCCACGCTGGGCATCGACTACGCCGAGTCCTCCTGC     300
clone  ATCCTGGCCATCATCCGCGCCATGTCCACGCTGGGCATCGACTACGCCGAGTCCTCCTGC     300
```

```
       ************************************************************

genome GCGGATGAAGGCCGGCTGCTGTTCAACCTGGCTGACTCCATCGAGGAGGGCACCATGCCC     360
clone  GCGGATGAAGGCCGGCTGCTGTTCAACCTGGCTGACTCCATCGAGGAGGGCACCATGCCC     360
       ************************************************************

genome CCCGAGCTGGTGAACTGCATCAAGAAGCTGTGGAAGGATGGGGGGGTCCAGGCGTGCTTT     420
clone  CCCGAGCTGGTGAACTGCATCAAGAAGCTGTGGAAGGATGGGGGGGTCCAGGCGTGCTTT     420
       ************************************************************

genome GACCGCGCTGCCGAGTATCAGCTCAACGACTCAGCTGCGTATTACCTGAACCAGCTGGAC     480
clone  GACCGCGCTGCCGAGTATCAGCTCAACGACTCAGCTGCGTATTACCTGAACCAGCTGGAC     480
       ************************************************************

genome AGGATCACAGCTGCCAACTACCTCCCCAACGAGCAGGACGTGCTGCGATCCCGAGTGAAG     540
clone  AGGATCACAGCTGCCAACTACCTCCCCAACGAGCAGGACGTGCTGCGATCCCGAGTGAAG     540
       ************************************************************

genome ACCACAGGGATCATCGAGACCAAGTTCTCTGTCAAAGACCTGAATTTCAGGATGTTTGAC     600
clone  ACCACAGGGATCATCGAGACCAAGTTCTCTGTCAAAGACCTGAATTTCAGGATGTTTGAC     600
       ************************************************************

genome GTGGGAGGGCAGCGCTCAGAGAGGAAGAAGTGGATCCACTGCTTCGAGGGGGTGACCTGC     660
clone  GTGGGAGGGCAGCGCTCAGAGAGGAAGAAGTGGATCCACTGCTTCGAGGGGGTGACCTGC     660
       ************************************************************

genome ATCATCTTCTGCGGGGCCCTGAGCGCCTACGACATGGTGCTGGTGGAGGATGATGAAGTG     720
clone  ATCATCTTCTGCGGGGCCCTGAGCGCCTACGACATGGTGCTGGTGGAGGATGATGAAGTG     720
       ************************************************************

genome AACCGGATGCATGAATCCCTGCACCTATTCAACAGTATATGCAACCACAAGTTCTTTGCT     780
clone  AACCGGATGCATGAATCCCTGCACCTATTCAACAGTATATGCAACCACAAGTTCTTTGCT     780
       ************************************************************

genome GCCACCTCCATCATCCTCTTCCTCAACAAGAAGGACCTTTTTGAGGAAAAGATCAAGAAA     840
clone  GCCACCTCCATCATCCTCTTCCTCAACAAGAAGGACCTTTTTGAGGAAAAGATCAAGAAA     840
       ************************************************************

genome GTTCATCTCAGCATCTGCTTCCCAGAGTATGATGGTCCAAACACGTTTGAGGACGCAGGG     900
clone  GTTCATCTCAGCATCTGCTTCCCAGAGTATGATGGTCCAAACACGTTTGAGGACGCAGGG     900
       ************************************************************

genome AATTACATCAAGACCCAGTTCCTGGACCTCAACATGAGGAAGGATGTGAAGGAGATCTAC     960
clone  AATTACATCAAGACCCAGTTCCTGGACCTCAACATGAGGAAGGATGTGAAGGAGATCTAC     960
       ************************************************************

genome AGCCACATGACCTGTGCCACAGACACGCAGAACGTCAAGTTCGTGTTCGACGCCGTCACA     1020
clone  AGCCACATGACCTGTGCCACAGACACGCAGAACGTCAAGTTCGTGTTCGACGCCGTCACA     1020
       ************************************************************

genome GACGTGATCATCAAAGAGAACCTCAAGGACTGTGGCCTCTTCTGA 1065
clone  GACGTGATCATCAAAGAGAACCTCAAGGACTGTGGCCTCTTCTGA 1065
       *********************************************
```

Figure S3. ErGNAT2 nucleotide sequence alignment. The reconstructed European robin ErGNAT2 sequence (Fig. S6) was aligned to the sequence derived from cloning from European robin retina cDNA (see Materials and Methods).

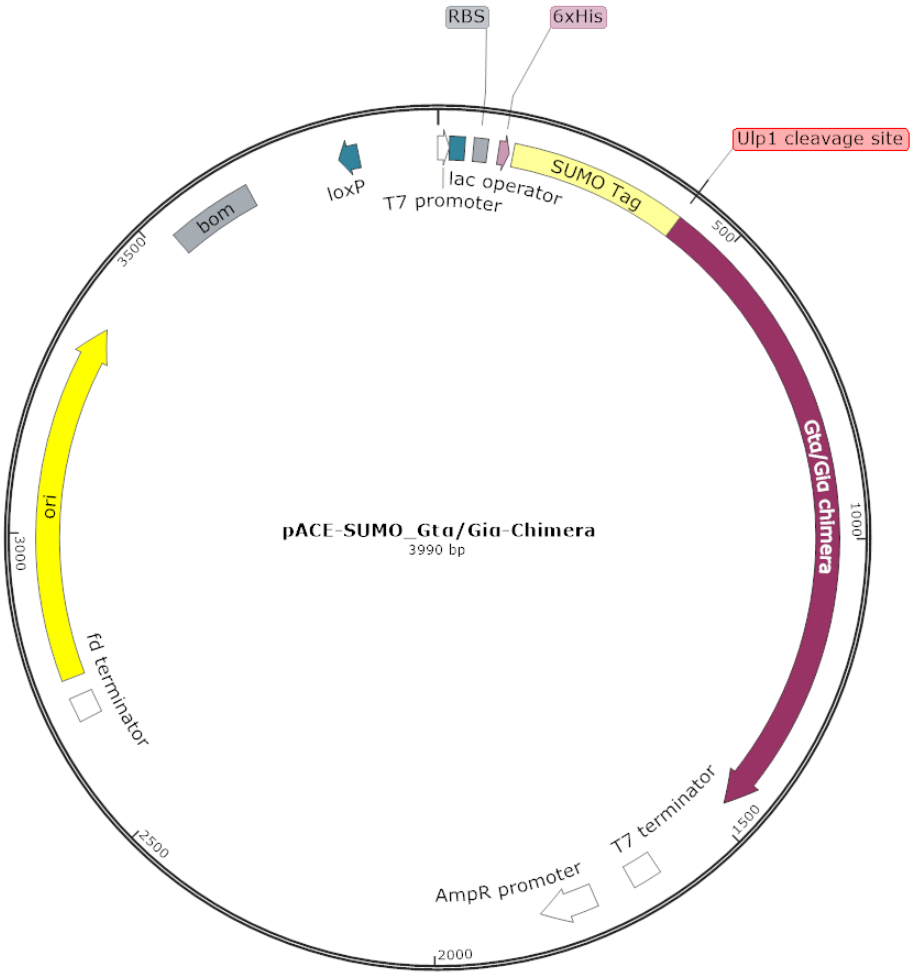

Figure S4. pACE-SUMO vector for Gtα/Giα chimera expression. Plasmid map was generated using SnapGene viewer. A commercial pACE vector (https://geneva-biotech.com/product_category/e-coli-cell-expression/multicoli/) was modified to contain a T7-lac expression cassette with a strong ribosome binding site and an N-terminal 6x histidine and Small Ubiquitin-like Modifier (SUMO) tag. Additional modifications include addition and removal of various restriction enzyme recognition sites.

A

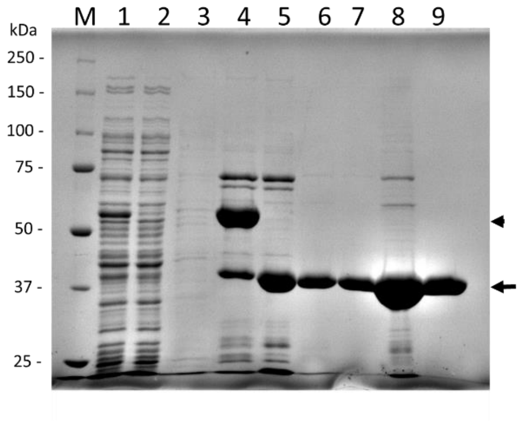

B

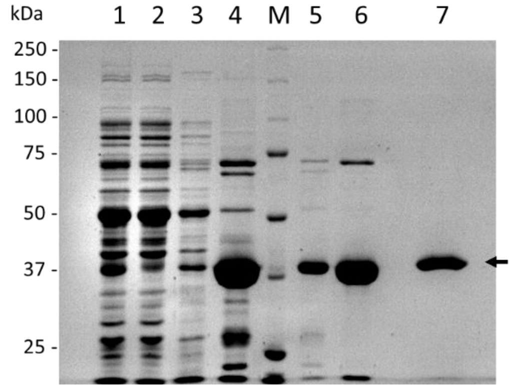

Figure S5. Expression and purification of the $G_t\alpha$/Giα chimera. Different steps yielding purified non-myristoylated (**A**) and myristoylated (**B**) $G_t\alpha$/Giα were analysed by sodium dodecylsulfate polyacrylamide gel electrophoresis (SDS-PAGE). (**A**) M, molecular mass marker; 1, soluble fraction of protein expression in E.coli that is applied on the Ni-NTA column; 2, nonbound fraction of Ni-NTA chromatography; 3, wash fraction; 4, elution form Ni-NTA column, arrow head at position of SUMO fusion construct; 5, digestion to remove the SUMO tag; 6, second Ni-NTA chromatography of $G_t\alpha$/Giα after digestion, nonbound fraction; 7, wash fraction; 8, fraction applied on the SEC column; 9, purified $G_t\alpha$/Giα after SEC, arrow.
(**B**) M, molecular mass marker; 1, soluble fraction of protein expression in *E.coli*.; 2, nonbound fraction of Ni-NTA chromatography; 3, Ni-NTA wash fraction; 4, Ni-NTA elution; 5, input fraction to IEC; 6, pooled fractions from IEC and applied to SEC; 7, representative collected SEC Fraction, arrow indicates purified Gtα/Giα.

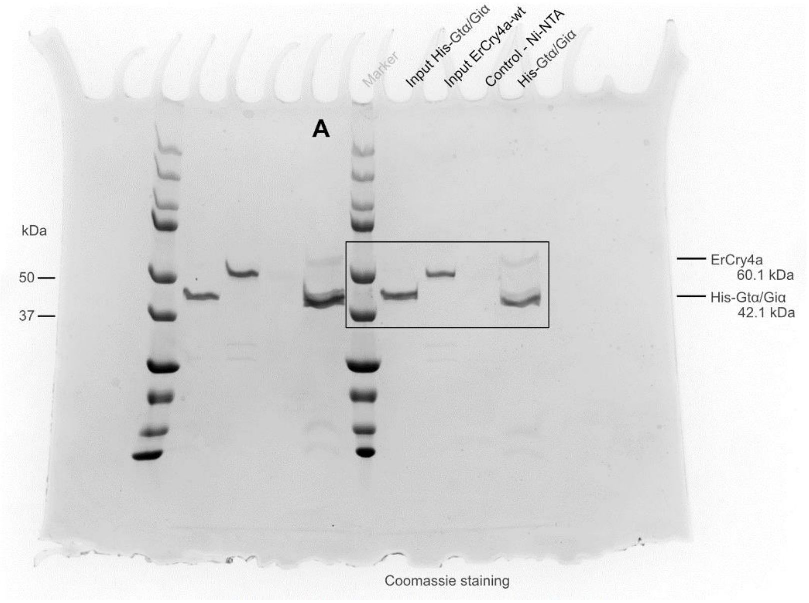

Figure S6. Full size image of pulldown experiment employing purified proteins shown in Fig.2 of the main text. Input lanes show the proteins used for the pulldown experiment. As a negative control only *Er*Cry4a, but no His-tagged $G_t\alpha$/Giα chimera, was incubated with a Ni-NTA affinity matrix. For the experiment, a pre-incubated mixture of both proteins was incubated with the matrix.

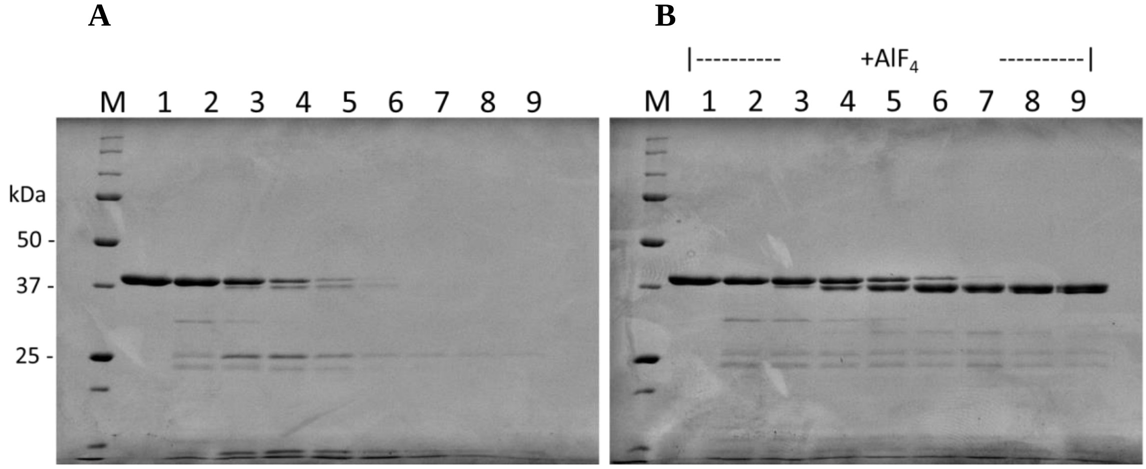

Figure S7. Limited proteolysis of Gtα/Giα in the absence or presence of AlF_4^-. (A) AlF_4^- absent (B) AlF_4^- Present. Analysis by SDS-PAGE. M – molecular mass marker in kDa, 1 – Sample before protease addition, 2 – Sample at 30s, 3 – Sample at 2 minutes, 4 – Sample at 5 minutes, 5 – Sample at 10 min, 6 – Sample at 20 min, 7 – Sample at 40 min, 8 – Sample at 60 min, 9 – Sample at 90 min.

Table S1. Primers for cloning steps

Primers for cloning into pFast:

ErCRY1a, ErCry1b (for) 5’GGGCGCCATGGGATCCATGGGGGTGAACGCCGTG3’
ErCRY1a (rev) 5’TACCGCATGCCTCGAGTTAATTTGTGCTCTGTCGCTGGAC3’
ErCRY1b (rev) 5’TACCGCATGCCTCGAGCTATTTTGATGTTTTGTCTGG3’
ErGNAT2chimera (for) 5’GGGCGCCATGGGATCCATGGGGAGCGGGGCCAGT3‘
ErGNAT2chimera (rev) 5’TACCGCATGCCTCGAGTCAGAAGAGGCCACAGTCC3‘

Primers for cloning of ErCry4a-497

Forward 5‘ – CTCGAGGATCCGAATTCAAG – 3‘
Reverse 5‘ – TTAGCGAGTCAGTTGTGCGG – 3‘

Primers for cloning of ErCry variants and constructs for FRET measurements

Primer Nr.	Sequence (5’→3’)
1 (for)	CTGGAAAGCGGCGGCGAAG
2 (rev)	GGCGGCGGTCACGAACTC
3 (for)	TTCGTGACCGCCGCCGGGATTACACATGGCATGGACGAGCTGTACAAGAGTGGATCCTCGGGATCATCAGGCGCGCCTATGCTGCATCGCACC
4 (rev)	GCCGCCGCTTTCCAGCTCGAGTTATTCTGTTGTTCGGGCCA
5 (for)	GTAAGGGCCCTATTCTATAGTGTCACC
6 (rev)	TCGAGAGGCGCGCCTGATGATCC
7 (for)	GGATCATCAGGCGCGCCTATGCTGCATCGCACCAT
8 (rev)	AGGGCCCTTACTCGAGCTCGAGTTATCTCGTGAGCTGG
9 (for)	TCTCGAGGTGTCTAAGGGCGAAGAG
10 (rev)	GGCGCGCCGTGGTGATGGTGATGATG
11 (for)	CTGGAAAGCGGCGGCGAAG
12 (rev)	GTGGTGATGGTGATGATGCATGGTGG
13 (for)	CATCACCATCACCACGGCGCGCCTATGGGGAGCGGGGC
14 (rev)	GCCGCCGCTTTCCAGCTCGAGGAAGAGGCCACAGTCCTTGAGG

Primers for cloning of Cone Transducin alpha and the Gtα/Giα chimera

ErGnat into pCold (for) 5‘-TTCCACTAGTATGGGGAGCGGGGC-3'
ErGnat into pCold (rev) 5‘-AAGGCTCGAGTCAGAAGAGCCGCAGTC-3'

Deletion 220-298 (for) 5'-GCAGGGAATTACATCAAGACCC-3'
Deletion 220-298 (rev) 5’ -CCAGTGGGGGAGCTTCGTC- 3’
Bovine 220-298 (for) 5’ -TTCGAGGGGGTGACCGCCATCATCTTCTGTGTGGCG- 3’
Bovine 220-298 (rev) 5’ -CTACATTAAGGGACGGAGAAGTATACACAAACTCGGAC-3’
Chimera into pET21a (for) 5’ -GGAGATATACATATGGGGAGCGGGGCCAGTG- 3’
Chimera into pET21a (rev) 5’ -GTGGTGGTGCTCGAGGAAGAGCCCGCAGTCCTTGAG- 3’
Chimera into SUMO (for) 5’ – CGTCGGATCCGGCGCCGGGAGCGGGGCCAGTGC – 3‘
Chimera into SUMO (rev)
5’ – GACTGGGAAAACCCTGGCGAGAATTCAGAAGAGCCCGCAGTCCTTG – 3‘
6AA deletion (for) 5‘ – GGGAGCGGGGCCAG – 3‘
6AA deletion (rev) 5‘ – ACCACCGATCTGTTCGC – 3‘
